# New data on the life cycle of *Nippostrongylus brasiliensis* (Travassos, 1914) (Nematoda: Heligmosomidae): development of eggs and larval stages in the intestine of naturally infected *Rattus norvegicus* (Berkenhout, 1769)

**DOI:** 10.1007/s00436-025-08462-8

**Published:** 2025-02-06

**Authors:** María Teresa Galán-Puchades, Mercedes Gómez-Samblás, María Trelis, Antonio Osuna, Rubén Bueno-Marí, Sandra Sáez-Durán, David Bruce Conn, Màrius V. Fuentes

**Affiliations:** 1 Parasites & Health Research Group, Department of Pharmacy, Pharmaceutical Technology and Parasitology, Faculty of Pharmacy, University of Valencia, Burjassot-Valencia, Spain; 2 Laboratory of Biochemistry and Molecular Parasitology, Institute of Biotechnology, University of Granada, Granada, Spain; 3 Laboratorios Lokímica, Paterna, Valencia Spain; 4 Department of Invertebrate Zoology, Museum of Comparative Zoology, Harvard University, Cambridge, MA USA; 5 One Health Center, Berry College, Mount Berry, GA USA

**Keywords:** *Nippostrongylus brasiliensis*, *Rattus norvegicus*, Natural life cycle, Egg embryonation, Intestinal larvae

## Abstract

*Nippostrongylus brasiliensis* is an intestinal parasite of rodents with a worldwide distribution. Due to the similarities between its life cycle and that of the human hookworms *Ancylostoma duodenale* and *Necator americanus*, *N. brasiliensis* has been widely used as a model in experimental research. We provide new data on the embryonation and hatching processes of *N. brasiliensis* eggs, not in the soil, but in the large intestine of naturally infected *Rattus norvegicus*. The parasite was investigated in 109 rats trapped in urban and periurban areas of Valencia, Spain. Rats were preserved at − 20 °C. After thawing, all the rat organs were examined. The contents of the large intestine were analysed by the Midi-Parasep® technique. *N. brasiliensis* (adult prevalence 69.72%) was identified by morphological and molecular techniques. Parasite eggs were found in 88.16% of the sediments. In addition to unembryonated eggs, eggs in different stages of embryonation were encountered in 67 of the 76 contents (88.16%). Free larval stages (L1 and more developed larvae) were also found in 47 of the 76 (61.84%) sediments studied. Filariform larval stages of *N. brasiliensis* were also sporadically detected in the lungs of the infected rats. According to our results, the development of eggs and larvae in the intestine of naturally infected rats opens up the possibility of autoinfection in the natural life cycle of *N. brasiliensis*, which could explain the high burdens of adult stages frequently found in the small intestine of infected rats.

**Supplementary Information:**

The online version contains supplementary material available at 10.1007/s00436-025-08462-8.

## Introduction

**Fig. 1:**
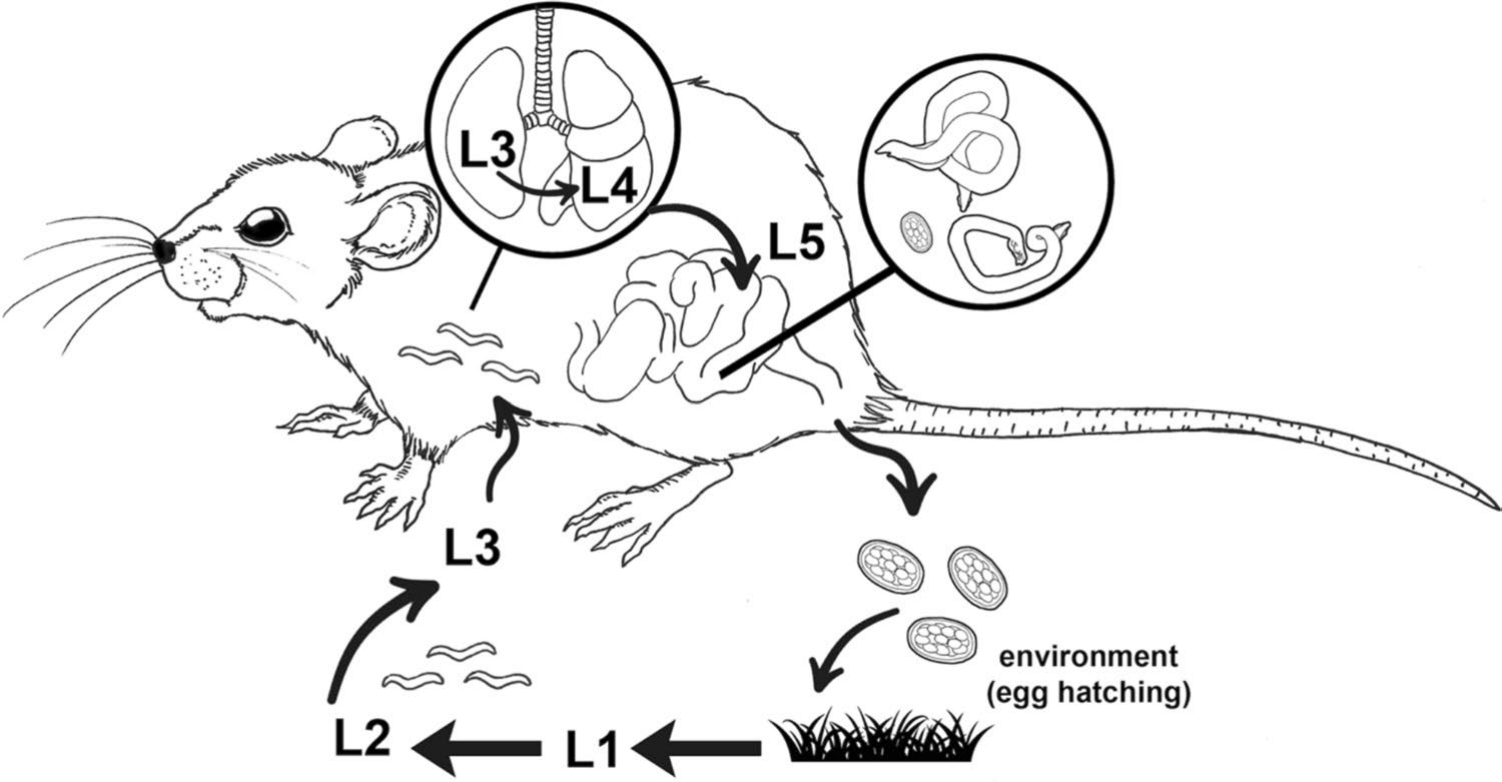
*Nippostrongylus brasiliensis* life cycle: unembryonated eggs shed in faeces mature and hatch in the soil. After two moults, L1 larvae develop into infective L3 which penetrate the skin of the rat. In the lungs, L3 turn into L4, which become adults in the intestine (L5) (illustration by Angela Debenedetti, PhD)

The nematode *Nippostrongylus brasiliensis* (Travassos, 1914) (= *Heligmosomum brasiliensis* Travassos 1914) is an intestinal parasite of rodents—primarily rats—with a worldwide distribution that was first described by Sadamu Yokogawa in 1920 in the Norway rat, *Epymis norvegicus* (= *Rattus norvegicus*) in Baltimore, USA, under the name of *Heligmosomum muris* Yokogawa 1920 (Yokogawa 1920). Just like the life cycle of hookworms, *N. brasiliensis* presents a direct or monoxenous life cycle. Females of this soil-transmitted helminth release immature eggs in the small intestine, which are shed in rat faeces. Rhabditiform larvae (L1) hatch in warm, moist soil, feed on soil bacteria and, after two moults, develop into infective strongyloid or filariform larvae (L3). L3 larvae infect new hosts mainly via the transcutaneous route, although the oral route is also possible. L3 carries out a somatic migration to the lungs, where they turn into L4 larvae, which, after their migration through the oesophagus, become adults in the small intestine (L5) (Yokogawa 1922) (Fig. 1).

Due to the similarities between the life cycle of *N. brasiliensis* and that of the human hookworms, *Ancylostoma duodenale* and *Necator americanus*, the rat nematode has been widely used as a model in experimental studies related to the biological characteristics of hookworms, its usefulness for vaccine candidates or also in studies of immunomodulation and types of immune responses against nematode infections (Camberis et al. 2003; Bouchery et al. 2017; Montaño et al. 2021; Thuma et al. 2022).

In the framework of a study on parasitic zoonoses of urban and periurban populations of *R. norvegicus* in the city of Valencia (Spain), we provide new data on the embryonation and hatching processes of *N. brasiliensis* eggs, not in the soil but in the large intestine of naturally infected Norway rats.

## Materials and methods

### Parasite investigation in the rats

Our research group signed an agreement with Valencia City Council, allowing us to investigate the presence of zoonotic parasites in rats trapped with snap traps by the pest control company Laboratorios Lokímica as part of the municipal pest control campaign in the city. Traps were checked every 1 to 2 days by the pest control personnel, and dead rats were then frozen at – 20 °C until parasitological examination.

After thawing, in addition to the examination of all the rat organs, the contents of the large intestine were also recovered. The Midi-Parasep® technique, which has proven to be a sensitive technique for concentrating helminth eggs and nematode larvae (Galán-Puchades et al. 2023), was used to concentrate the intestinal contents. Part of the sediment obtained in the Midi-Parasep® was frozen at − 80 °C for subsequent molecular analysis to investigate the presence of protists, while the other part was preserved in 10% formalin to investigate the presence of resistant forms of parasites, mainly helminth eggs or larval stages as found in the case of rats infected by *Angiostrongylus cantonensis* (Galán-Puchades et al. 2023). No other larval stages were expected to be found.

Four drops (each of approximately 0.03 mL volume) of the sediments were observed between the slide and coverslip under the microscope at × 100 and × 400 magnifications.

### *Nippostrongylus brasiliensis* identification

*Nippostrongylus brasiliensis* adult specimens were morphologically identified between slide and coverslip with lactophenol, a clearing agent, based on data from previous reports published by specialists (Yokogawa 1920; Kassay 1982).

For molecular identification, total genomic DNA was extracted separately from several adult nematodes (males and females), using the DNeasy Blood & Tissue kit (Qiagen, Cat. No. 69504), following the manufacturer’s instructions. The lysis step was performed overnight at 56 °C. Total DNA from the larval stages was also isolated from the faeces using FavorPrep Stool DNA Isolation Mini kit (Favorgen, Cat. No. FASTI001). Nematode species identity was confirmed by PCR and sequencing of the internal transcribed spacer region (ITS-1, 5.8S and ITS-2) using the forward primer NC5: 5′-GTAGGTGAACCTGCGGAAGGATCATT-3′ and reverse primer NC2: 5′-TTAGTTTCTTTTCCTCCGCT-3′ (Zhu et al. 2000; Madrid et al. 2012). Cytochrome c oxidase 1 (COX1) was also amplified (own designed forward primer NB_48F: 5′-AAGTTCCAATCATAAGGATATTG-3′ and reverse primer HC02198: 5′-TAAACTTCAGGGTGACCAAAAAATCA-3′) based on Folmer et al. (1994).

The PCR product was sequenced by Sanger sequencing at the Institute of Parasitology and Biomedicine “López-Neyra” (Granada, Spain) genomic facilities. The sequences were quality-trimmed and analysed by Geneious software [10.1093/bioinformatics/bts199].

The sequences were aligned with GenBank sequences for the ITS and COX1 genes using Geneious alignment software. A phylogenetic tree was then constructed using MrBayes version 3.2.6, following the methodology outlined by Tian et al. (2023), applying Bayesian inference (Supplementary Figs. 1 and 2). The GTR substitution model was applied to ensure the best fit. *Heligmosomoides polygyrus* (KJ994540) was selected as the outgroup for COX1, while *Vexillata liomyos* Mexico (MN366464) served as the outgroup for ITS1.

### Statistical analysis

The analysis of *N. brasiliensis* parasitation was carried out by calculating the prevalence, mean abundance, median intensity and range (Bush et al. 1997). The comparison of the prevalences between intrinsic (age and sex) and extrinsic (trapping site and season of capture) factors was made through the *χ*^2^ test, and the comparison of the mean abundance by means of standard non-parametric tests, i.e., the Mann–Whitney (*U*) and Kruskal–Wallis (*H*) tests. Statistical significance was established at *p* < 0.05.

## Results

The presence of *N. brasiliensis* was investigated in 109 *Rattus norvegicus* (47 males, 57 females, 5 indet.). According to their body weight, external morphometry and reproductive status (Gosálbez 1987), 69 rats were considered adults (≥ 150 g), 38 juveniles (< 150 g), and 2 indet*.* Rats were trapped in 15 of the 19 districts into which Valencia is divided. Three trapping sites are under continuous pest control, namely the sewer system (58 rats were obtained), parks and gardens (36 rats) and periurban orchards (15 rats). The rats studied were trapped in spring (*n* = 61), autumn (*n* = 18) and winter (*n* = 30).

We found adult stages of *N. brasiliensis* in the small intestine of the rats as well as different types of eggs in the contents of the large intestine, both unembryonated and embryonated. We also found different larval stages in the large intestine of the studied individuals as well as larvae in the lungs of several rats.

### Molecular identification

Molecular identification determined by BLAST analysis confirmed that the adults in the small intestine as well as the resistant forms in the large intestine contents (eggs and larval stages) belonged to *N. brasiliensis* species. The obtained sequences for the mitochondrial COX1 region and the ITS1 were 437 bp and 551 bp in length, respectively. These sequences have been deposited in the GenBank database under accession numbers PP389490, PP389491, and PP389492. Prior to this study, no *N. brasiliensis* sequences had been reported from Spain. Therefore, comparisons were made with the available sequences of *N. brasiliensis* in the GenBank database, specifically from Japan, New Zealand, the USA, and France.

The Bayesian algorithm emerged as the optimal method for resolving nematode phylogenies. Two phylogenetic trees were constructed using Bayesian inference, revealing that our samples clustered together in the same clade. This clustering demonstrated the phylogenetic placement of *N. brasiliensis* isolates relative to other nematode species (Supplementary Figs. 1 and 2).

### Adult stages

**Fig. 2:**
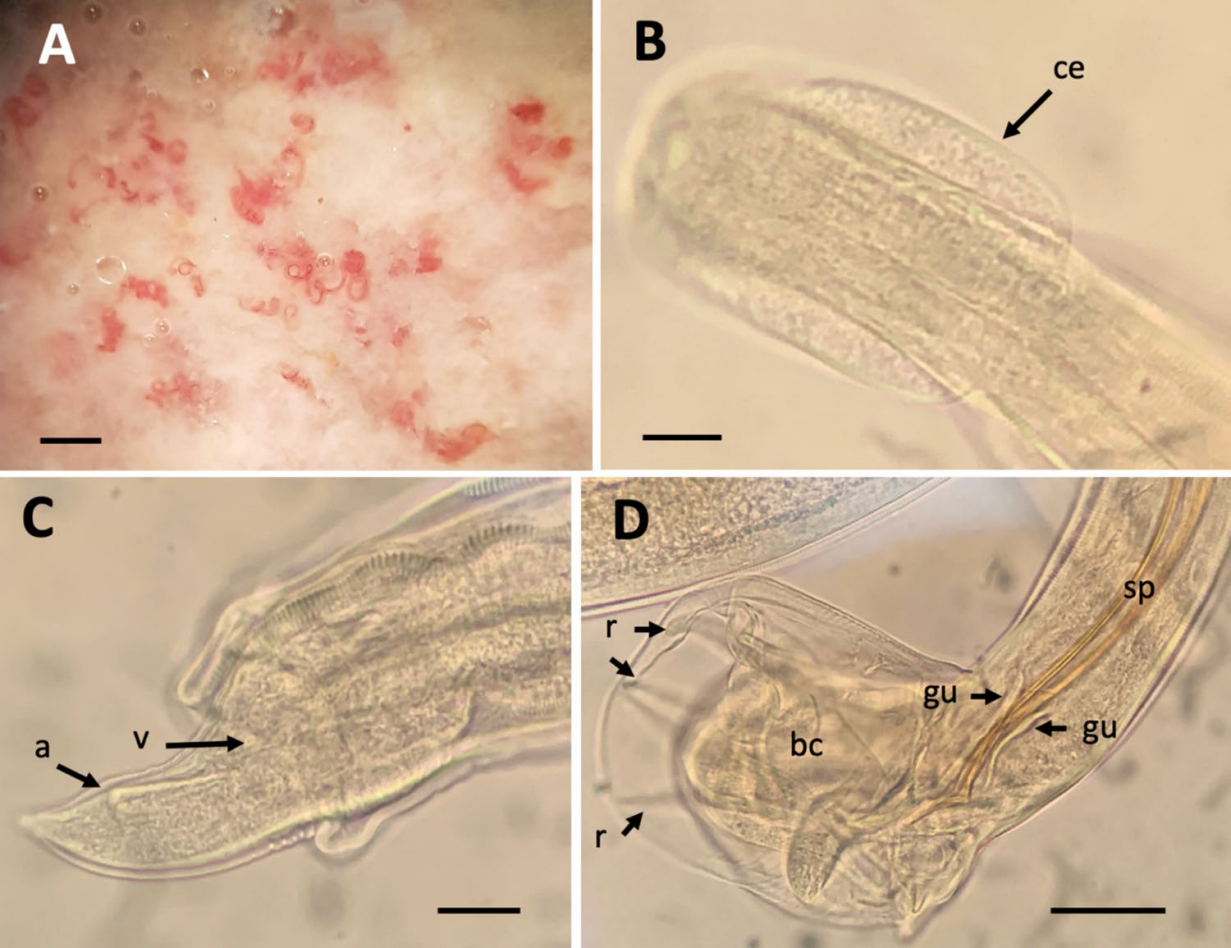
*Nippostrongylus brasiliensis* adult stages: a segment of the small intestine of a *R. norvegicus* specimen containing coiled individuals (**A**). Details of the cephalic expansion (ce) in **B**, and in **C** the posterior end of a female individual with the arrows pointing at the anus (a) and vulva (V). In **D**, posterior end of a male showing the copulatory bursa (bc) with the rays (r), the two brown sclerotized spicules (sp), and the two sclerotized gubernacula (gu). Scale bars: **A**, 1 mm; **B**, 10 µm; **C**, **D**, 50 µm

Adult males and females of *N. brasiliensis* were found in 76 of the 109 rats studied (prevalence 69.72%) (Fig. 2). Table 1 lists the measurements of both male and female individuals of the nematode. The parasite was found in all 15 districts where the rats were obtained as well as in the three types of trapping sites.

**Table 1:** Measurements in µm of the intestinal adult males and females of *Nippostrongylus brasiliensis*

Variables	Males (*n* = 50)	Females (*n* = 50)
Length	3200–4000	3500–5500
Width	59–100	75–150
Oesophagus length	285–355	310–470
Spicule length	505–545	–
Spicule length	505–545	–
Gubernaculum length	20–48	–
Distance vulva-posterior end	–	100–130
Distance anus-posterior end	–	60–78

*n*, number of individuals studied

Table 2 shows the prevalence, mean abundance and median intensity of *N. brasiliensis* adults in the 109 hosts studied according to their sex, age, and site and season of capture. There were no statistical differences in the prevalence of the parasite regarding the sex, the age of the rats or the season of capture. However, there were statistically significant differences when comparing the prevalences obtained in the sewer system and parks and gardens (*χ*^2^ = 7.443; *p* = 0.0043) and between the sewer system and orchards (*χ*^2^ = 4.266; *p* = 0.0346). Likewise, *N. brasiliensis* was found in rats captured in the three trapping seasons, although no statistical differences were found among the seasons.

**Table 2:** Descriptive statistics of *Nippostrongylus brasiliensis* in the 109 *Rattus norvegicus* individuals analysed

Independent variables	*n*	Prevalence (%) (95% CI)	Mean abundance (SE)	Median intensity (range)
Host sex				
Males	36	76.6 (64.5–88.7)	188.5 (40.3)	246.1 (1–1,000)
Females	38	66.7 (54.5–78.9)	118.3 (48.8)	177.5 (1–2,536)
Host age				
Juveniles	26	68.4 (53.6–83.2)	149.5 (70.9)	218.5 (1–2,536)
Adults	50	72.5 (62.0–83.0)	146.1 (30.0)	201.7 (1–1200)
Sites of capture				
Sewer system	48	82.8 (73.1–92.5)	233.7 (54.9)	5.1 (1–25)
Parks and gardens	20	56.5 (40.3–72.7)	57.7 (16.5)	18.64 (1–91)
Orchards	8	53.3 (28.1–78.5)	9.1 (4.7)	10.79 (1–111)
Season of capture				
Spring	42	68.3 (56.6–80.0)	139.1 (31.4)	202.1 (1–1,200)
Autumn	12	66.7 (44.9–88.5)	69.6 (39.4)	104.3 (2–700)
Winter	22	73.3 (57.5–89.1)	200.9 (90.0)	274.0 (1–2,536)

*n* number of infested hosts, *CI* confidence interval, *SE* standard error

In terms of the mean abundance [total mean abundance was 144.6 (31.0)], there were statistical differences with respect to the site of capture, being higher in the sewer system (*H* = 17.33; *p* < 0.001). The total median intensity was 207.5, with a range of 1–2,536.

### Eggs

**Fig. 3:**
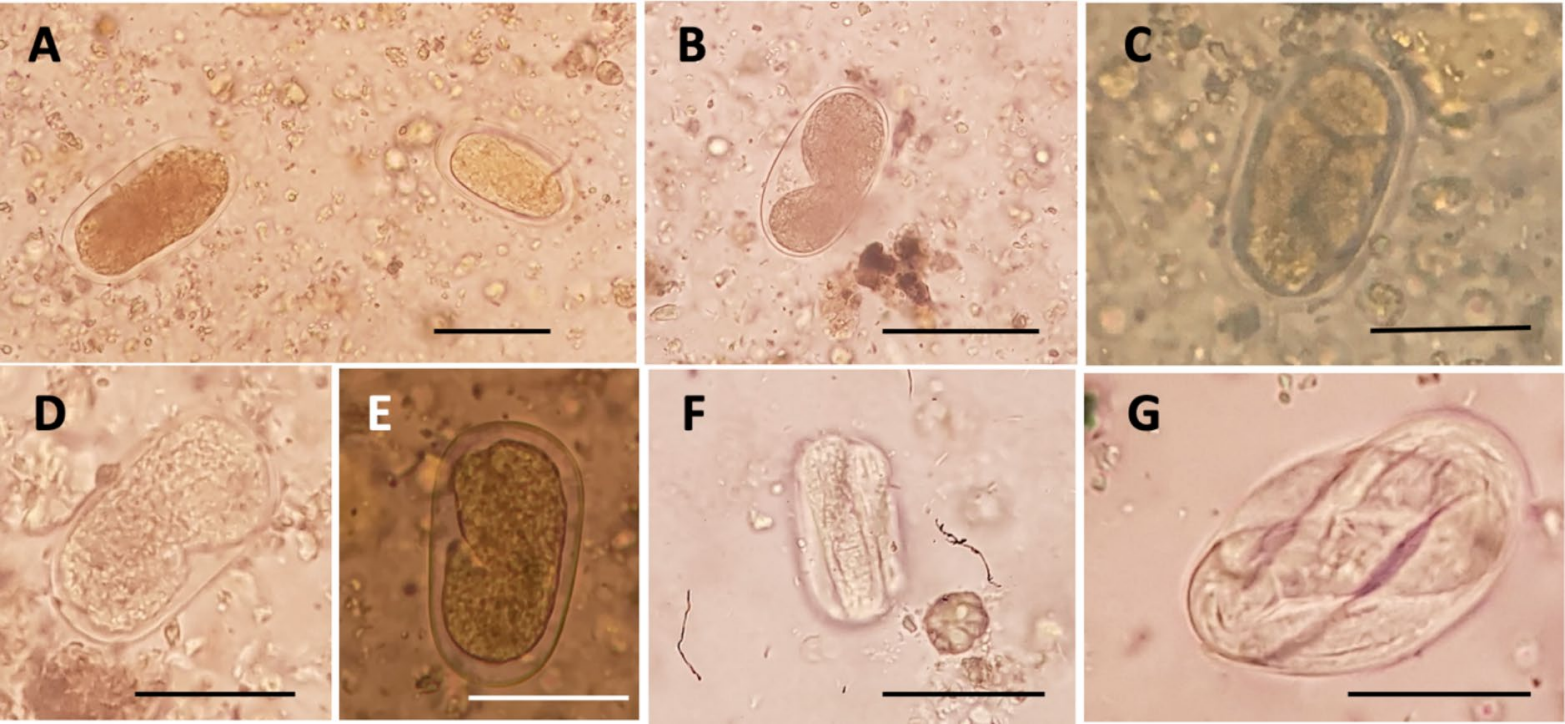
*Nippostrongylus brasiliensis* eggs: unembryonated in **A**; with two-blastomere (**B**) and four-blastomere embryos (**C**); with early comma stage (**D**) and tadpole stage embryo (**E**). Larvated egg with pretzel L1 stage embryo in F and egg with fully developed L1 larva in **G**. Scale bars: 50 µm

*Nippostrongylus brasiliensis* eggs were found in 67 of the 76 sediments studied (88.16%).Different types of eggs were detected, presenting an average size of 55.2 × 30.8 µm (43.5–68.3 × 25.3–38 µm; *n* = 30) (Fig. 3). Unembryonated eggs were found in 65 of the 76 sediments (Table 3). Two types of unembryonated eggs could be distinguished. We found the typical brown ellipsoidal thin-shelled eggs in the early morula stage (Fig. 3A left). In addition, unembryonated but yellowish eggs containing a zygote were also encountered in most of the sediments studied (Fig. 3A right). These eggs are usually slightly smaller than the brownish ones.

**Table 3:** Number of parasitized rats (N) and prevalences (%) of the evolutive stages of *Nippostrongylus brasiliensis* found in the large intestine of the 76 infected rats

**Evolutive stages**	***N***	**%**
UNe	65	85.53
Ee	67	88.16
UNe + Ee	65	85.53
L	47	61.84

*UNe* unembryonated eggs, *Ee* embryonated eggs, *L* larval stages

**Fig. 4:**
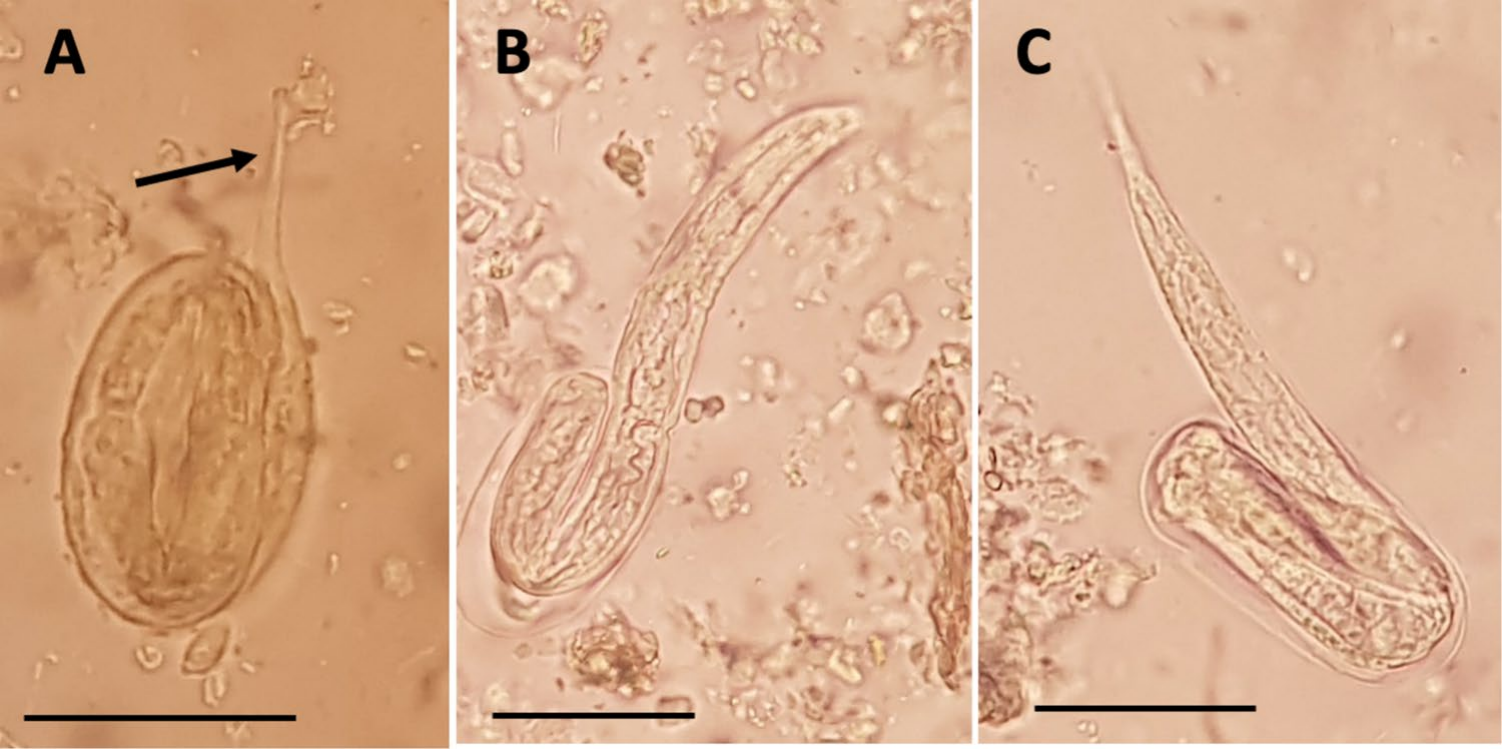
Hatching of *Nippostrongylus brasiliensis* eggs: the arrow in **A** points at the tail of the emerging larva. The L1 larva is almost released from the egg, emerging the head first in **B** and the tail in **C**. Scale bars: 50 µm

In addition to the unembryonated eggs, eggs in different stages of embryonation were found in 67 of the 76 large intestine contents studied (Table 3). Both types of eggs, embryonated and unembryonated, were found in 65 of the 76 sediments. According to the nomenclature by Conn (2000), we detected eggs with two- and four-blastomere embryos (Fig. 3B, 3C, respectively), as well as eggs with embryos in the early comma stage (Fig. 3D), and in the tadpole stage (Fig. 3E). Larvated eggs containing pretzel L1-stage embryos (Fig. 3F) and fully developed first-stage larvae (L1) were also found (Fig. 3G). Figure 4 shows the hatching of eggs releasing the L1 which were also encountered in the sediments. Larger brown eggs were found only in the early morula stage or in the early comma stage.

We observed that the hatching mechanism of the eggs of *N. brasiliensis* is like that of *Ancylostoma ceylanicum* and *A. tubaeforme*, i.e., the larva perforates the shell with its sharp tail before reversing to emerge head first (Fig. 4A, 4B), or as well as that of *Haemonchus contortus* and *Heterodera* (Mkandawire et al. 2022), in which the larva perforates the egg shell with its tail which emerges first (Fig. 4C).

### Intestinal larval stages

**Fig. 5:**
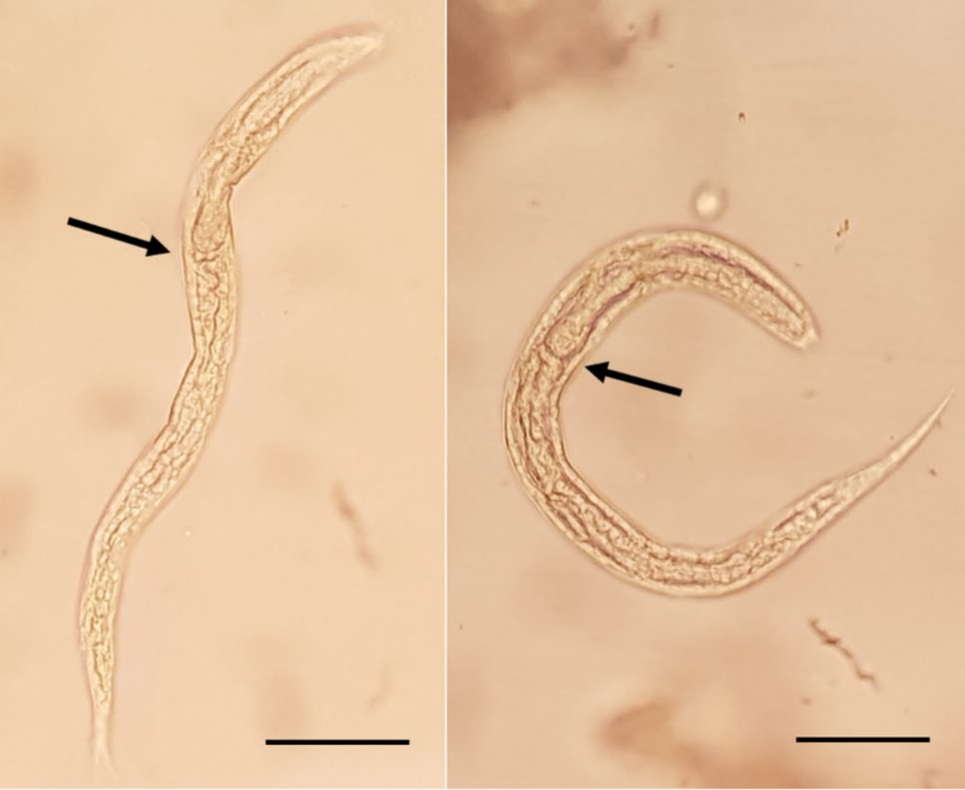
*Nippostrongylus brasiliensis* L1 larvae: arrows point at the end of the rhabditiform oesophagi. Scale bars: 50 µm

**Fig. 6:**
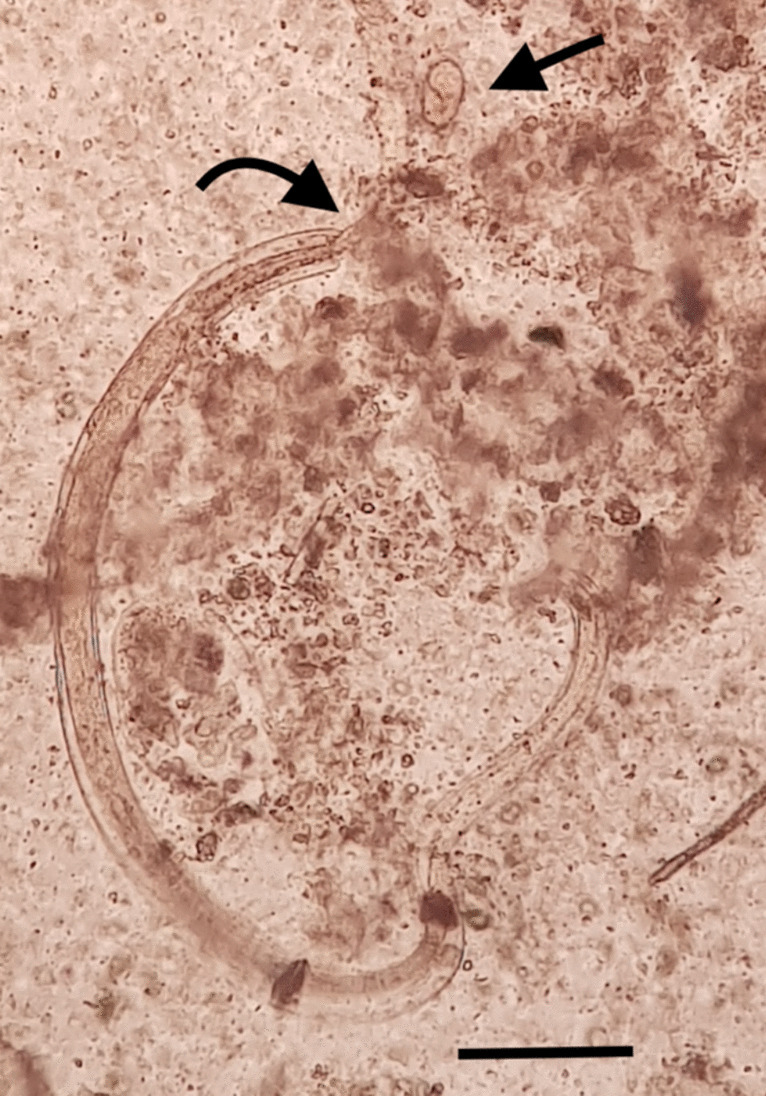
More developed *Nippostrongylus brasiliensis* larval stage: one arrow points at an egg and the other at the larva emerging from the sheath. Scale bar: 100 µm

Free larval stages of *N. brasiliensis* were also found in at least (as only four drops were analysed) 47 of the 76 sediments studied (Table 2). L1 larvae were detected in 46 sediments (60.53%) (Fig. 5). The average size of the larvae was 227.4 × 12.9 µm (201.3–294 × 12.7–15.2 µm; *n* = 30). The rhabditoid oesophagus (ES) was 72 µm long (65.8–81; *n* = 8). Four more developed larval stages were also encountered in three of the sediments studied (Table 2). Specifically, larval sizes were 401.8 × 17.7 µm (ES, 121.4 µm long), 540 × 15.2 µm (ES, 245 µm), 686 × 20.2 µm (ES, 188.3 µm) and 780 × 27.3 µm (ES, 180.5 µm). One of these longer larvae was in the process of moulting (Fig. 6).

### Pulmonary larval stages

**Fig. 7:**
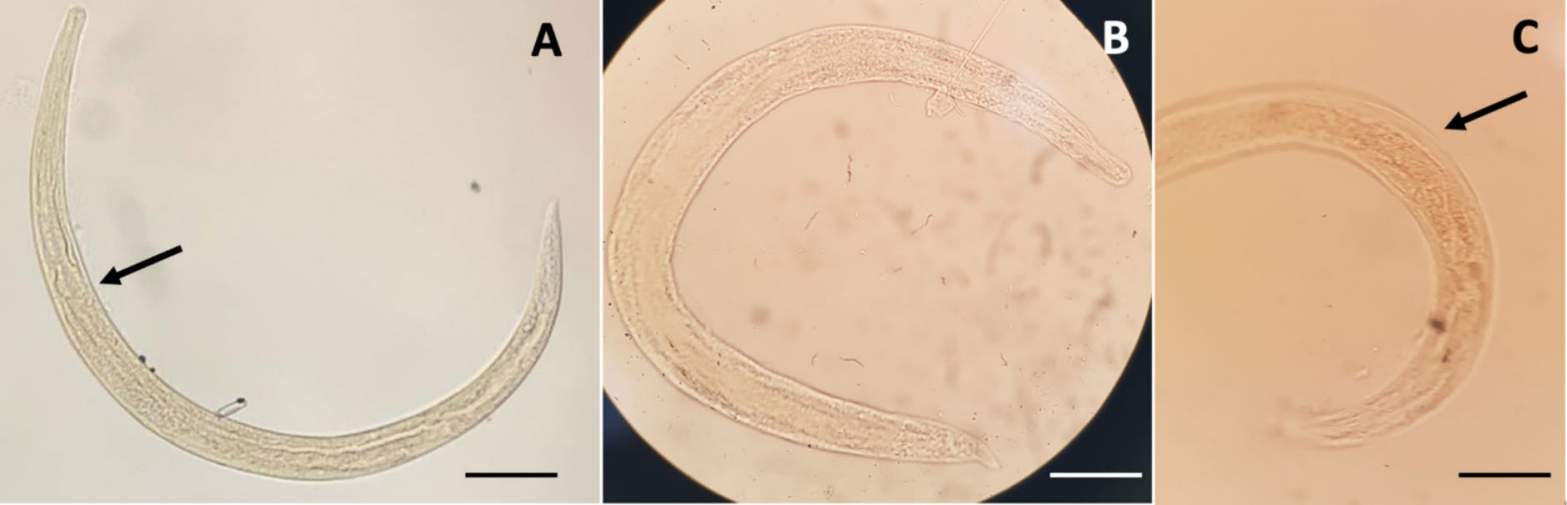
Pulmonary *Nippostrongylus brasiliensis* larvae: the arrow in **A** points at the end of the filariform oesophagus and the sheath in **C**. Scale bars: 50 µm

Filariform larval stages of *N. brasiliensis* were also sporadically found in the lungs of the infected rats, specifically in 7 rat individuals (9.21%) (Fig. 7). The smallest sizes found were two larvae measuring 531.6 × 29.3 µm (ES, 175 µm) and 686 × 32.9 µm (ES, 187.2 µm). The largest larvae measured 708 × 29–1127 × 50.6 µm (873.1 × 45 µm, *n* = 7). The oesophagi were 172- to 239-µm long (203.8 µm).

## Discussion

A high prevalence of *N. brasiliensis* was found in the studied rats, close to 70%, and no statistical differences were found according to the sex or the age of the rats, or the season of capture. Only rats in the sewers showed higher prevalences than those obtained in parks and gardens and orchards. Likewise, the mean abundance was also higher in the sewers. A favourable microclimate as well as the higher rat density in sewer systems (Pascual et al. 2020) could lead to a higher concentration of infective larval stages in the soil, which favours *N. brasiliensis* transmission.

This is the first time that embryonation and hatching of *N. brasiliensis* eggs and larval development are reported, not in the soil, but in the large intestine of the hosts.

The size range of the unembryonated eggs of *N. brasiliensis* in the environment is 54.60–61.88 × 30.9–34.58 µm (Yokogawa 1922; Camberis et al. 2003). The size of the eggs found in the sediments in this study was 43.5–68.3 × 25.3–38 µm. Therefore, in addition to eggs with the same range of dimensions as those found in the environment, smaller eggs were also detected in the large intestine as well as larger eggs corresponding to those ready for hatching, including a fully developed first-stage larva.

There are several data on the size of the larval stages of *N. brasiliensis* in the literature. Reports on the first-stage larvae in the environment measuring 280–300 × 15–18 µm and also L1 with a length of 270–550 µm have been published (Anderson 2000). As for the size of the second-stage larvae (L2), a size of 470–750 µm was reported (Anderson 2000). In this study, smaller larvae were found in the intestinal contents (201.3–294 × 12.7–15.2 µm). However, we also found larvae measuring 401.8–780 × 17.7–27.3 µm in the large intestine. Considering that in this study larvae measuring 531.6 and 686 µm were found in the lungs and that the infective L3 larvae are those that reach the lungs and develop into larger L4 larvae, this means that L1 can even develop into infective L3 in the large intestine of rats naturally infected by *N. brasiliensis.*

Measurements of 500–600 × 20–25 µm (Nembo et al. 1993), 500–700 µm (Anderson 2000) and 620–750 µm (Camberis et al. 2003) were reported for the infective larvae. Therefore, the largest larvae we found in the lungs would correspond to the more developed L4 larvae, the stage which leaves the lungs to develop into the adult stage in the small intestine.

It should be noted that in our study, we did not analyze the intestinal contents immediately after the death of the rats. Traps were checked every 1 to 2 days by the pest control personnel and dead rats were then frozen. It is difficult to ascertain how much time elapsed between the death of the *R. norvegicus* individuals and subsequent freezing. It could be several hours or even more than 24 h. However, most of the rats were in good conditions at the time of dissection after thawing, which is likely to indicate that most of them had been dead for a short time in a city with warm temperatures that would favor rapid decomposition. Therefore, there are two options: that the subsequent development of the eggs and larval stages occurred exclusively by the passage of time in the dead rats or that this development may take place when the rats are alive that could lead to autoinfection.

With respect to the hatching of nematode eggs, it is accepted that for most parasitic nematodes the factors which trigger egg eclosion remain unknown (Mkandawire et al. 2022). Particularly, in the case of hookworms, whose egg hatching occurs in the soil just as in the case of *N. brasiliensis* eggs, several environmental factors have always been considered determinants for their eclosion, namely an optimal pH, temperature and oxygen availability, carbon dioxide levels, as well as the texture, moisture, and microbial activity of the soil. However, for the eggs we found, not in the environment but in the intestinal content, the intrinsic and/or extrinsic factors which triggered their embryonation and hatching are, logically, unknown. If the embryonation/eclosion of the eggs were exclusively due to the passage of time in the dead rats we analysed, that would mean that time would be a significant factor triggering egg development, the reason why the phenomenon may not occur in live rats. However, a common symptom of intestinal helminth infections, such as constipation, would be enough to provoke this development of eggs and larval stages in the large intestine of the infected rats. According to the experiments conducted by Yokogawa (1922), eggs hatch rapidly within 12–15 h, although in 20–24 h most of the unembryonated eggs develop into first stage larvae and hatch. However, these data are not known in nature, and therefore the embryonation period could be longer or perhaps even shorter.

Practically, and to the best of our knowledge, since Yokogawa’s studies in the 1920s, and those of Haley in the 1960s, there have been no further data on the *N. brasiliensis* life cycle (Haley 1962). Camberis et al. (2003) also described the cycle with the same characteristics as the previous data. In fact, there are many previous studies dealing with *N. brasiliensis,* but in an experimental way, i.e., from the point of view of its role as a model of immune response to human hookworms. The remaining studies citing *N. brasiliensis* in nature do not involve any investigation of its life cycle. Most researchers extract the adult stages of the parasite directly from the small intestine or investigate the presence of eggs in faeces and almost never investigate the content of the large intestine as we did (Waugh et al. 2006; Gómez Villafañe et al. 2008; Coomansingh and Sharma 2009; Kataranovski et al. 2011; Panti-May et al. 2015; Chaudhary et al. 2016; Carvalho-Pereira et al. 2018; Galán-Puchades et al. 2018; Hancke and Suarez 2018; Rara et al. 2023).

To date, there have apparently not been any previous reports of *N. brasiliensis* eggs hatching in the intestine of their hosts in nature*.* However, recently, in a study on urban/agricultural rodents in the Philippines (Matullano et al. 2024), the authors found not only adult *N. brasiliensis* in the small intestine of the animals studied, but they also detected fully embryonated eggs containing the L1 larvae as well as unidentified nematode larval stages in the intestinal contents—different from the *A. cantonensis* larvae they identified—and which could correspond to the larval stages of *N. brasiliensis*.

An environment heavily contaminated with L3 larval stages could also explain the high burdens frequently found in the small intestine of the rats, but not the presence of developed eggs or larval stages in the large intestine. Therefore, based on our results, it is worth investigating whether autoinfection plays a role in the natural life cycle of *N. brasiliensis*.

The fact that we found large numbers of adult stages of *N. brasiliensis* in the small intestine of most infected rats—with cases of thousands of adult stages—might support this possibility. Furthermore, some other authors also found high burdens of *N. brasiliensis* in the intestine of naturally infected rats (Gomez Villafañe et al. 2008; Panti-May et al. 2015; Carvalho-Pereira et al. 2018; Rara et al. 2023).

In this context, under normal circumstances, *Strongyloides* infections in rodents do not lead to autoinfection (Breloer and Abraham 2017). However, alterations in the host immune response may promote this phenomenon (Nutman 2017). In this regard, none of our studied rats was infected by *N. brasiliensis* only*.* The parasitofauna of the 76 rats consisted of 16 species, 4 protists, and 12 helminths (cestodes, nematodes and acanthocephalans) (unpublished data). The rats were coinfected with 3 to 13 species (mean 6.2 species/rat) and surely with bacteria and viruses. Therefore, coinfections could modulate the rat’s immune system in the sense of favouring autoinfection by *N. brasiliensis*.

The phenomenon of autoinfection is a strategy used by parasites to increase their transmission success and also the fitness and life history attributes of those parasites that are capable of autoinfection are immediately improved (Zimmermann et al. 2015). In the case of *N. brasilienses*, autoinfection will lead to a considerably increase in the number of reproductive stages of the parasite in the small intestine of the host. In fact, we found hundreds and hundreds of adult stages in the small intestine of several individuals of the studied hosts. Consequently, the more adult stages the more eggs will be shed to the outside world, thus increasing the likelihood of transmission to another host in a potentially hostile environment.

Finally, in most of the aforementioned studies reporting the presence of *N. brasiliensis*, the species is usually the most prevalent parasite and often with high parasite intensities. These facts could be consistent with a successful autoinfection strategy carried out by the parasite.

We are aware that it is difficult to corroborate whether autoinfection could occur in the natural life cycle of *N. brasiliensis* in hosts coinfected with other parasites since validating this possibility in nature would require experimental replication in the laboratory using the same strain of *N. brasiliensis* and in coinfected rats with similar biological and immunological characteristics to those we have studied. However, despite the complexity of the study, parasite concomitant infections are experimentally studied (Cox 2001; Behnke et al. 2001; Bouchery et al. 2017; Murambiwa et al. 2020). Therefore, in addition to further studies of the intestinal contents of rats immediately after death, challenging experimental studies would be necessary to understand the extent of this possible pattern in the natural life cycle of *N. brasiliensis*.

## Supplementary Information

Below is the link to the electronic supplementary material.

Supplementary file1 Phylogenetic tree of *Nippostrongylus brasiliensis* (Valencia, Spain) based on COX1 partial gene sequences. *Heligmosomoides polygyrus* (KJ994540) as outgroup (PDF 94 KB)

Supplementary file2 Phylogenetic tree of *Nippostrongylus brasiliensis* (Valencia, Spain) based on ITS1 partial gene sequences. *Vexillata liomyos* Mexico (MN366464) as outgroup (PDF 92 KB)

## Data Availability

No datasets were generated or analysed during the current study.
