# Supplementary figures and images for: New data on the life cycle of Nippostrongylus brasiliensis (Travassos, 1914) (Nematoda: Heligmosomidae): development of eggs and larval stages in the intestine of naturally infected Rattus norvegicus (Berkenhout, 1769)
Source: Parasitol Res. 2025 Feb 6;124(2):20. doi: 10.1007/s00436-025-08462-8 (PMC11799119; doi:10.1007/s00436-025-08462-8)

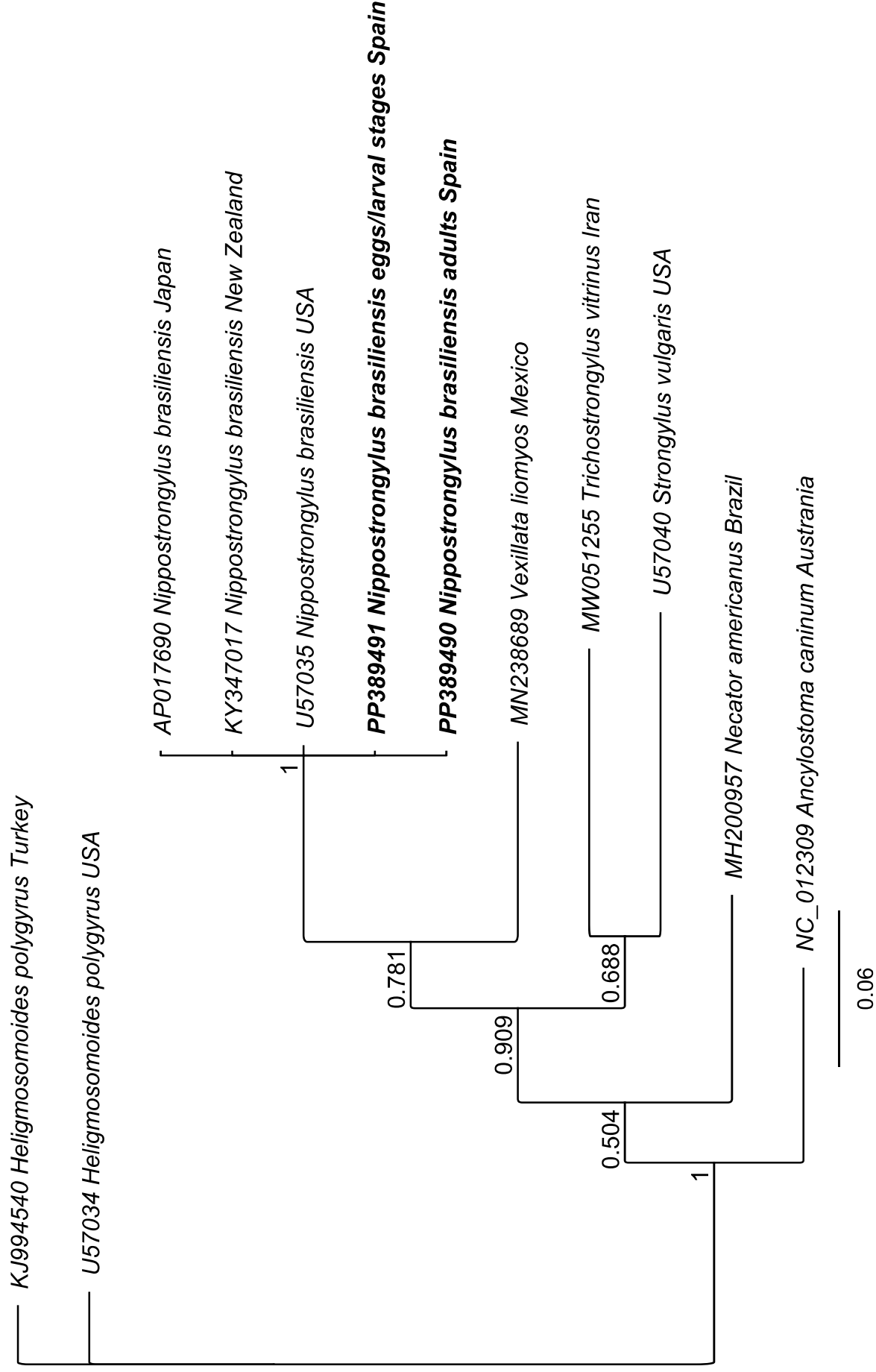

Supplement: Supplementary file 1 — Supplementary file1 Phylogenetic tree of Nippostrongylus brasiliensis (Valencia, Spain) based on COX1 partial gene sequences. Heligmosomoides polygyrus (KJ994540) as outgroup (PDF 94 KB) [file 436_2025_8462_MOESM1_ESM.pdf]

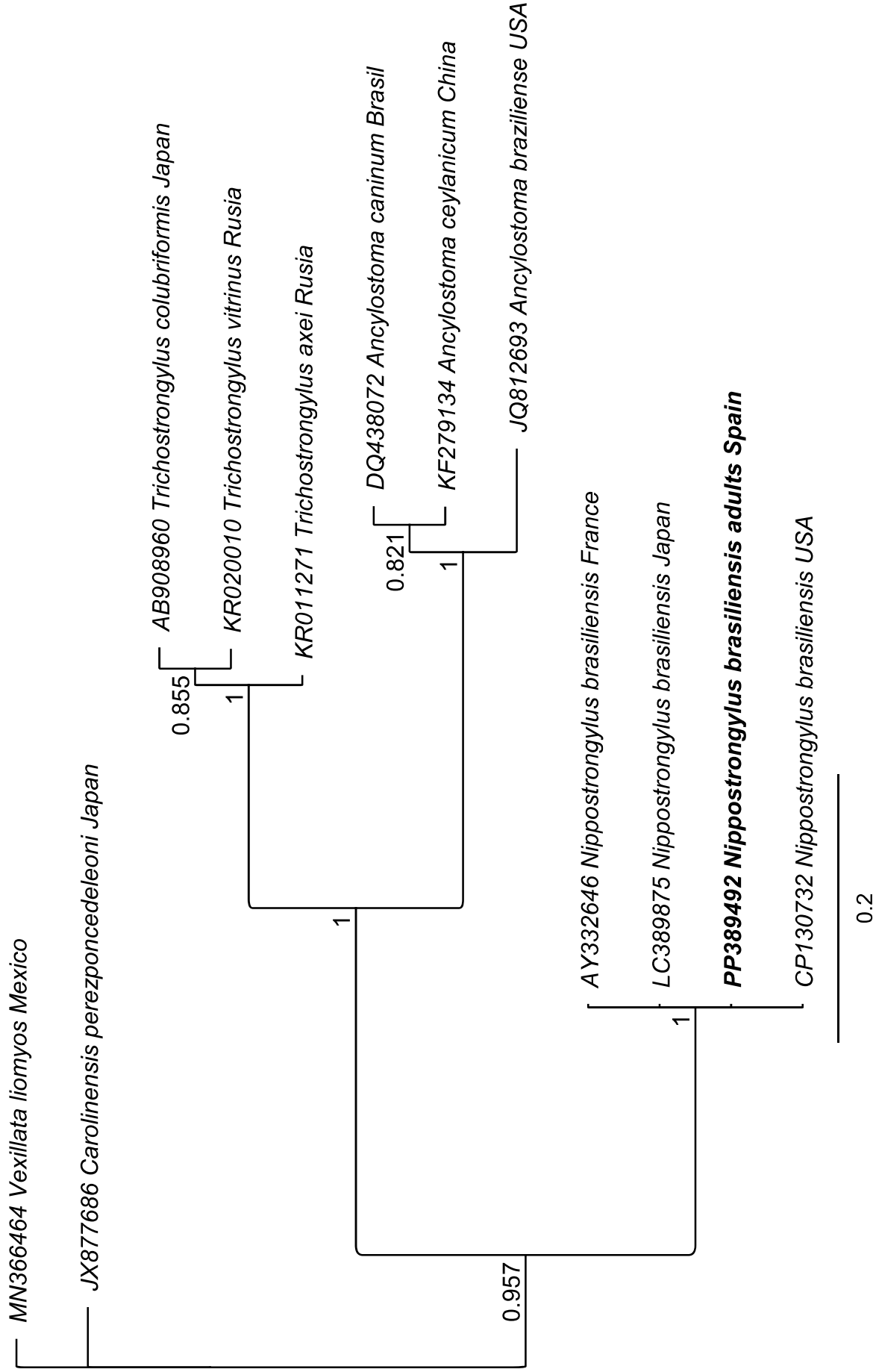

Supplement: Supplementary file 2 — Supplementary file2 Phylogenetic tree of Nippostrongylus brasiliensis (Valencia, Spain) based on ITS1 partial gene sequences. Vexillata liomyos Mexico (MN366464) as outgroup (PDF 92 KB) [file 436_2025_8462_MOESM2_ESM.pdf]
